# Mechanical Thrombectomy Complicated by Pulmonary Artery Pseudoaneurysm Treated With Endovascular Thrombin Injection

**DOI:** 10.7759/cureus.63688

**Published:** 2024-07-02

**Authors:** Alexandra So, Richard Krauthamer

**Affiliations:** 1 Department of Medicine, University of California San Francisco, San Francisco, USA; 2 Department of Radiology, Torrance Memorial Medical Center, Torrance, USA

**Keywords:** endovascular, embolization, thrombectomy, pseudoaneurysm, pulmonary embolism

## Abstract

Pulmonary thrombectomy for acute pulmonary arterial embolism has emerged as an effective endovascular therapy for high-risk patients. While the procedure is typically performed safely and efficaciously, given the nature of the treatment, complications may rarely occur. Pulmonary pseudoaneurysm is a rare condition occurring in a variety of settings, including pulmonary thrombectomy. To our knowledge, we present the first published case of pulmonary artery pseudoaneurysm following percutaneous thrombectomy, along with its successful endovascular management.

## Introduction

Pulmonary embolism is the leading cause of in-hospital mortality and the third most frequent cause of cardiovascular death [1,2]. While traditionally pulmonary embolism therapy has consisted of anticoagulation, thrombolysis, or surgery, numerous percutaneous interventional technologies have been developed with potential utility in patients with intermediate-high-risk or high-risk pulmonary embolism [1,3,4]. These technologies include catheter-directed thrombolysis and aspiration thrombectomy. These interventional treatment options offer the benefit of potential expedited improvement in right ventricular function and hemodynamics [1,5,6]. However, further investigation is warranted to identify optimal patients.

While these procedures can be safely performed in the majority of cases, complications can rarely occur, including pulmonary pseudoaneurysms. To the best of our knowledge, we present the first case of pulmonary arterial mechanical thrombectomy complicated by pseudoaneurysm formation that was subsequently managed successfully with endovascular intra-arterial injection of thrombin.

## Case presentation

**Figure 1:**
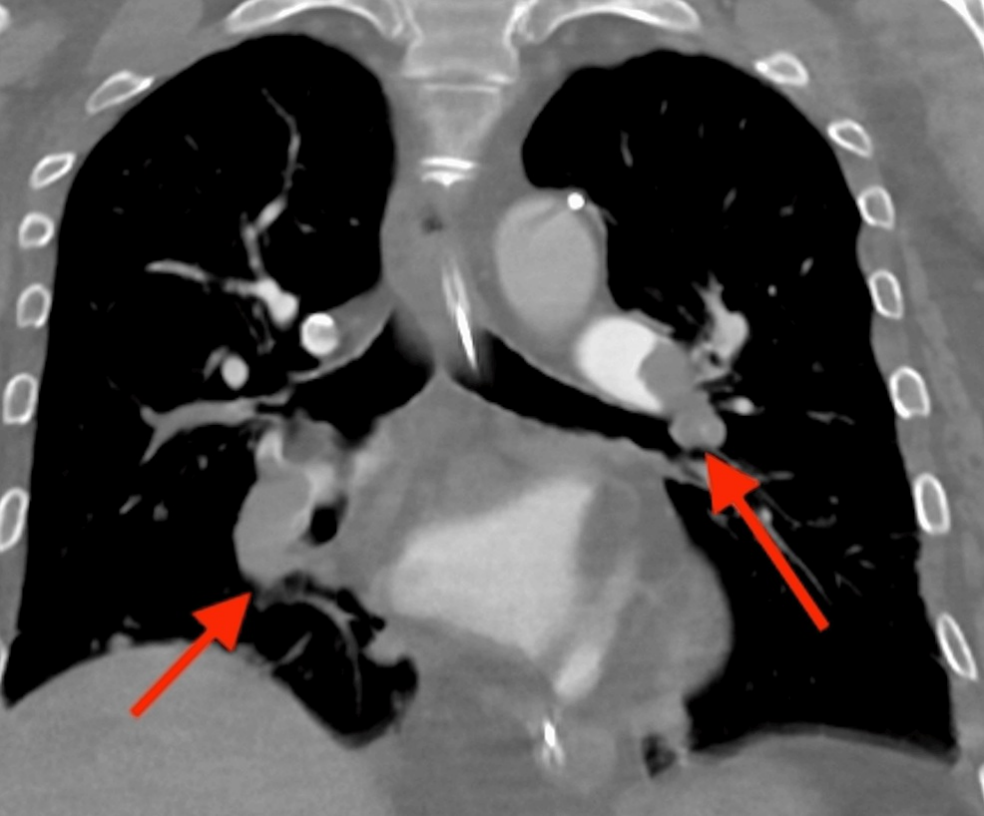
A CT pulmonary angiogram upon initial presentation demonstrates left main pulmonary arterial and right lower lobar thromboembolism (red arrows) CT: computed tomography

The patient provided written consent to the publication of this case. A 77-year-old female patient with a past medical history of dementia, hypertension, hypothyroidism, recent myxedema coma, and biopsy-proven pulmonary carcinoid was initially hospitalized following an episode of altered mental status. Her condition improved following intravenous levothyroxine, and she was discharged to an inpatient rehabilitation facility. One day later, during physical therapy, she experienced sudden hypoxic acute respiratory failure with a temporary loss of pulses. The return of spontaneous circulation was promptly achieved following a brief round of chest compressions, and she was admitted to the intensive care unit. A CT pulmonary angiogram demonstrated bilateral main pulmonary arterial thromboembolism extending to subsegmental branches (Figure 1). Although a CT head scan was negative following a fall she had three days prior, a high-dose bolus of TPA was not administered due to the risk of occult bleeding. Given her underlying massive pulmonary embolism, she was referred for an emergent mechanical thrombectomy. She was intubated pre-procedurally for acute respiratory failure.

**Figure 2:**
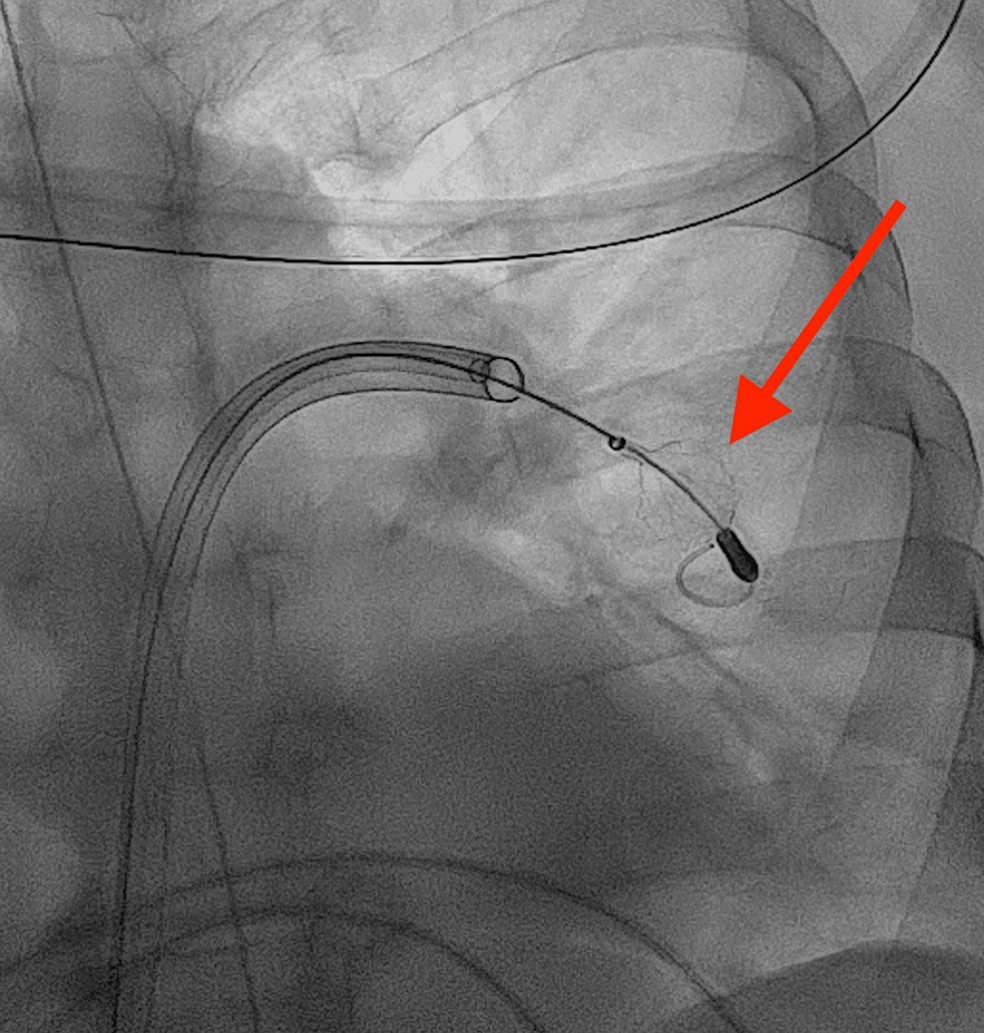
A disk-technology-based FlowTriever 2 catheter (red arrow) was deployed in the left pulmonary artery to disrupt adherent clots and facilitate subsequent aspiration

Following sequential right common femoral venous and pulmonary arterial catheterization, mechanical aspiration thrombectomy was performed with the FlowTriever system (Inari Medical, Irvine, California), which has been previously described in detail [7]. Following heparinization to achieve an activated clotting time of 240 seconds, a right pulmonary arterial mechanical thrombectomy was performed using the 24-French Triever24 aspiration catheter. A substantial thrombus was aspirated, with improvements in systemic and pulmonary arterial blood pressures. Left pulmonary arterial mechanical thrombectomy was then performed initially using the Triever24 and curved 20-French Triever20 aspiration catheters without significant clot removal. A disk-technology-based FlowTriever 2 catheter was then deployed to disrupt adherent clots and facilitate subsequent aspiration (Figure 2).

**Figure 3:**
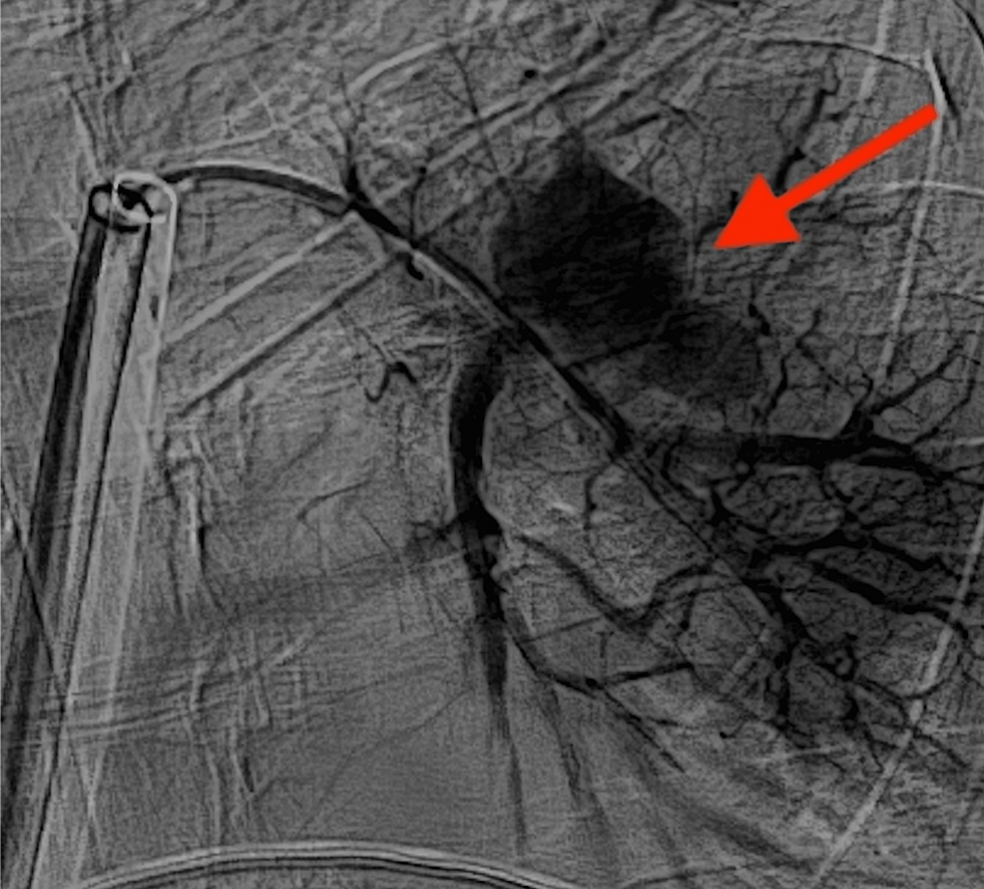
Left pulmonary angiography after thrombectomy demonstrated a bilobed central left pulmonary artery pseudoaneurysm (red arrow)

**Figure 4:**
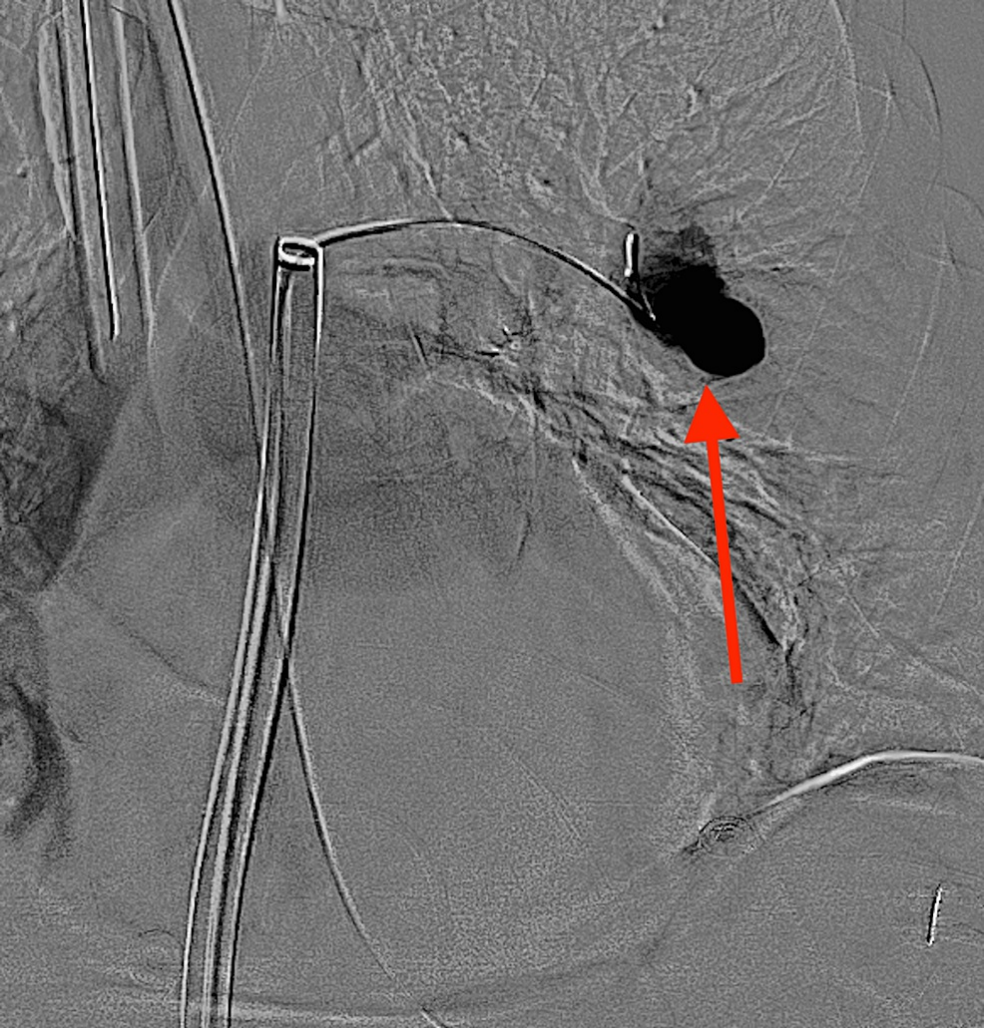
Microcatheter and microwire combination used to cannulate the pseudoaneurysm (red arrow) prior to thrombin injection

**Figure 5:**
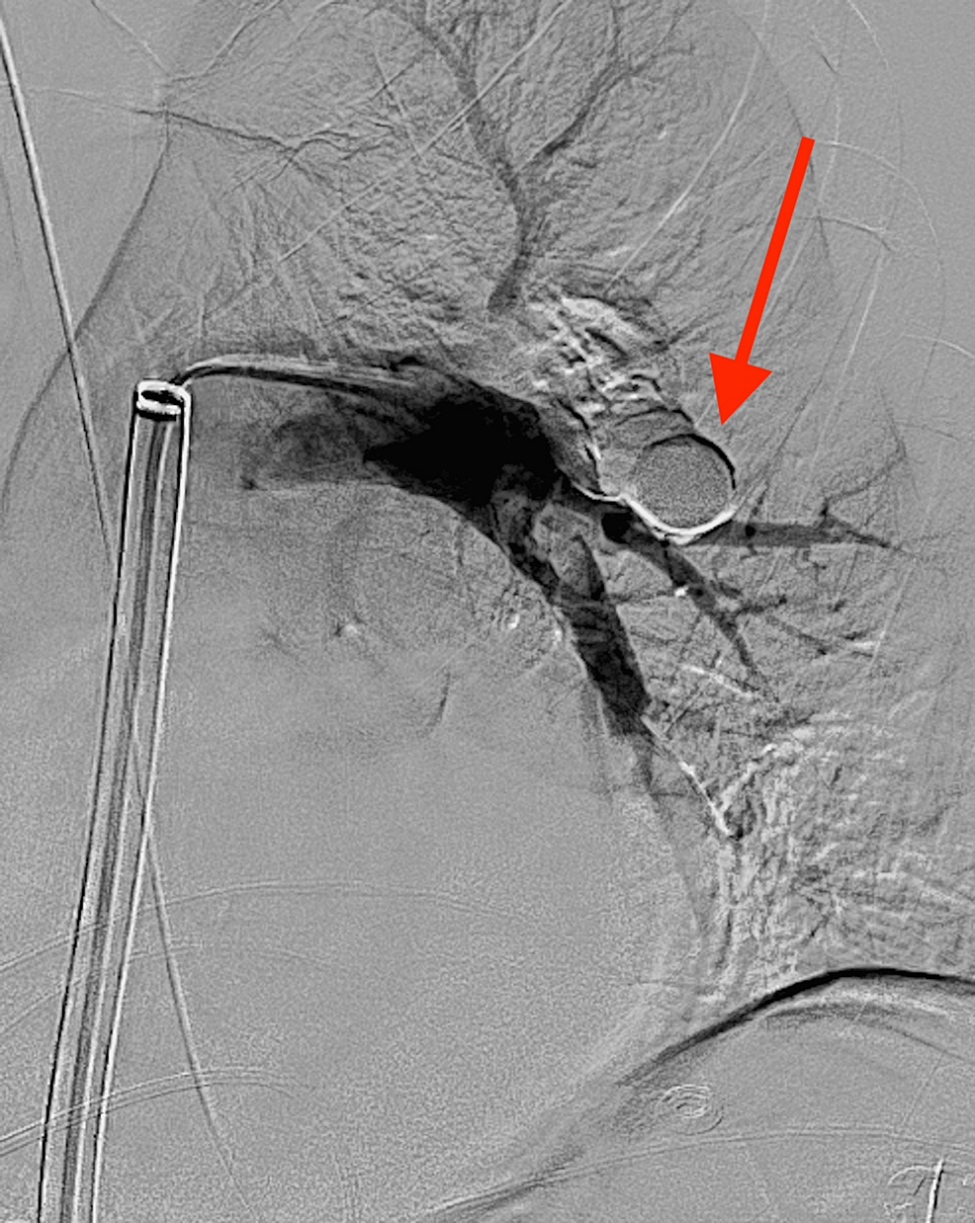
Final left pulmonary angiogram demonstrated complete pseudoaneurysm thrombosis without further enhancement (red arrow)

**Figure 6:**
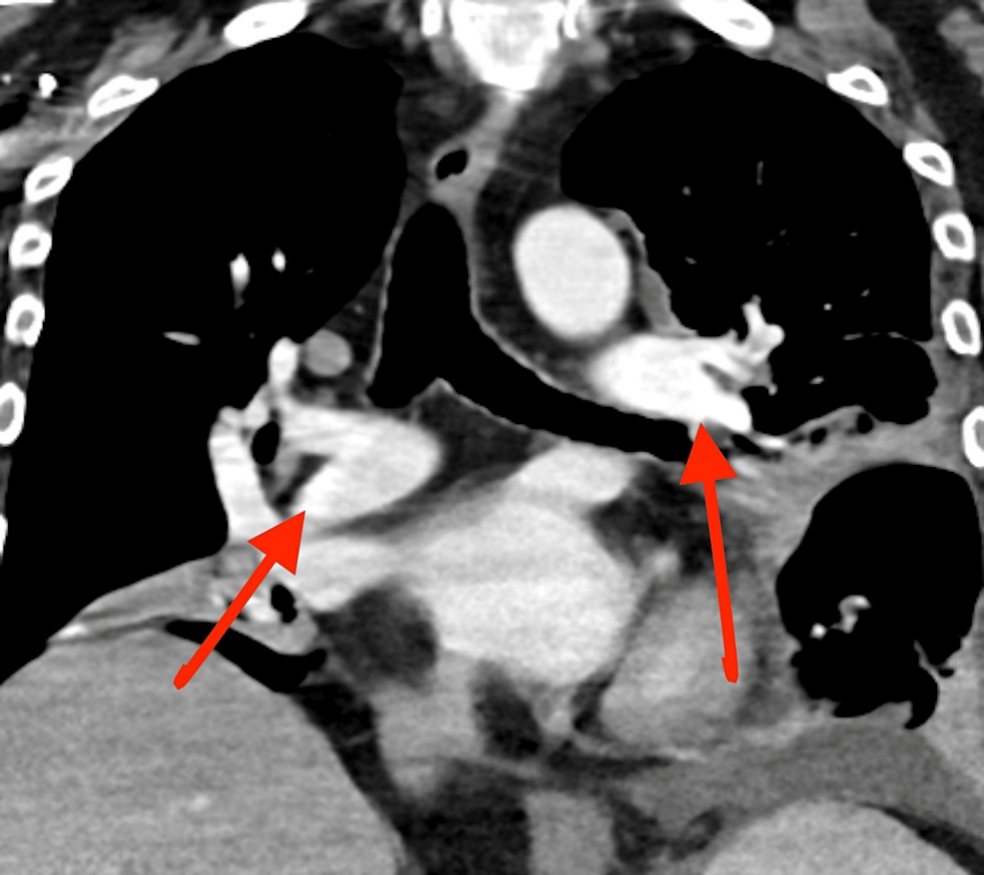
Repeat CTA of the chest one month following thrombectomy demonstrated resolution of previously identified pulmonary clot burdens (red arrows)

At this time, a large quantity of blood was suctioned from the endotracheal tube by the anesthesiologist. Repeat left pulmonary angiography demonstrated a bilobed central left pulmonary artery pseudoaneurysm (Figure 3). Protamine 50 mg was administered intravenously to reverse anticoagulation. The endotracheal tube was redirected into the right mainstem bronchus to protect the airways from additional aspirated blood. A SwiftNINJA 2.4-French microcatheter (Merit Medical, South Jordan, Utah) and Fathom-16 microwire (Boston Scientific, Marlborough, Massachusetts) were utilized to canulate the pseudoaneurysm (Figure 4). Upon confirmation of positioning within the pseudoaneurysm, 5000 units of bovine thrombin (Pfizer, Manhattan, New York) were injected slowly. The final left pulmonary angiogram revealed complete pseudoaneurysm thrombosis (Figure 5), and the patient’s hemoptysis resolved. Given the overall improvement in hemodynamics and to prevent subsequent re-hemorrhage, additional thrombectomy was deferred and pulmonary arterial catheters were withdrawn. A retrievable IVC filter was placed prophylactically. The patient remained stable without any recurrence of hemoptysis, and one month later, prior to discharge, a repeat CTA of the chest revealed normalization of the right-to-left ventricular ratio without evidence of the left pulmonary artery pseudoaneurysm or significant residual pulmonary embolus (Figure 6).

## Discussion

Pulmonary pseudoaneurysms are rare but can be seen in a variety of settings, including infections, vasculitis, trauma, lung cancer, and iatrogenic causes such as mechanical thrombectomy or Swan-Ganz catheter manipulation [8]. While mortality approaches 50%, treatment strategies include surgery, embolization, and stent graft placement [9]. Iatrogenic pulmonary artery pseudoaneurysms can have a range of outcomes, ranging from asymptomatic behavior to death via exsanguination. As a result, awareness of this rare but potential complication as well as familiarity with potential strategies to manage this outcome are of critical importance when performing pulmonary arterial thrombectomy.

Pulmonary artery pseudoaneurysms were traditionally managed with surgery, but endovascular repair has emerged as the preferred option due to its minimally invasive nature [10]. Furthermore, catheter-directed angiography also allows a definitive diagnosis of pseudoaneurysms, including those that may not be detected on CTA [9,10]. A review of the literature demonstrates the utilization of multiple endovascular treatment strategies, including stent grafting [11,12], placement of Amplatzer septal occlusion devices (Abbott, Irvine, CA) [13], and embolization with a variety of agents, including detachable balloons and coils, polyvinyl alcohol, and liquid embolic agents (including Onyx (Medtronic, Irvine, CA), Squid (Balt, Irvine, CA), and n-butyl cyanoacrylate (Cerenovus, Fremont, CA)) [10,14-17]. Further analysis of the literature demonstrates coil and microcoil embolization to be the most frequently utilized form of management for endovascular pseudoaneurysm embolization, whether performed proximally only, proximally and distally, or intra-aneurysmal [10,14-17]. Pseudoaneurysm exclusion with a stent-graft or Amplatzer septal occluder is likely optimal for more proximal or main pulmonary artery pseudoaneurysms, in contrast to segmental or more distal pseudoaneurysms, such as in the presented case. While coil embolization was considered in our case, this strategy was deemed to be potentially time-consuming as well as carrying the risk of permanent distal non-target embolization. Thrombin injection offers the potential advantage of rapid occlusion, which is particularly beneficial given the high risk of mortality from this condition. It could also offer the possibility of maintaining the persistence of the parent vessel.

As demonstrated in our case, pulmonary pseudoaneurysm thrombin injection may be a preferred endovascular method due to rapid occlusion, safety, and efficacy. Open surgical repair could be an alternative technique, but it would carry a high risk of significant morbidity and mortality. Overall, the wide variety of endovascular management agents reflects the complex nature of this condition, and the choice of embolization strategy will vary by case and pseudoaneurysm location. Performing thrombectomy procedures with anesthesia when available is recommended, as is having thoracic surgical support if needed. Continuing to compile further case series and performing larger-scale clinical trials would be beneficial to further refine endovascular management strategies.

## Conclusions

To our knowledge, we present the first case of pseudoaneurysm in the setting of mechanical thrombectomy of pulmonary arterial thromboembolism, as well as its successful catheter-directed management utilizing intra-arterial injection of thrombin. After consideration of the potential methods to manage this unexpected and sudden complication during emergent pulmonary arterial thrombectomy, we chose endovascular treatment given the benefits of a minimally invasive technique. Furthermore, given the proven efficacy of thrombin injection in managing pseudoaneurysms, this agent was chosen for embolization. Novel endovascular therapy devices for the treatment of pulmonary embolism continue to emerge and have revolutionized the treatment of this potentially fatal and devastating condition. However, future clinical studies should continue to identify optimal patients for treatment as well as potential complications and their subsequent management techniques.

## References

[1] Götzinger F, Lauder L, Sharp AS (2023). Interventional therapies for pulmonary embolism. Nat Rev Cardiol.

[2] Jiménez D, Bikdeli B, Barrios D (2018). Epidemiology, patterns of care and mortality for patients with hemodynamically unstable acute symptomatic pulmonary embolism. Int J Cardiol.

[3] Klok FA, Piazza G, Sharp AS (2022). Ultrasound-facilitated, catheter-directed thrombolysis vs anticoagulation alone for acute intermediate-high-risk pulmonary embolism: Rationale and design of the HI-PEITHO study. Am Heart J.

[4] Sanchez O, Charles-Nelson A, Ageno W (2022). Reduced-dose intravenous thrombolysis for acute intermediate-high-risk pulmonary embolism: rationale and design of the pulmonary embolism international thrombolysis (Peitho)-3 trial. Thromb Haemost.

[5] Giri J, Sista AK, Weinberg I (2019). Interventional therapies for acute pulmonary embolism: current status and principles for the development of novel evidence: a scientific statement from the American Heart Association. Circulation.

[6] Stevens SM, Woller SC, Baumann Kreuziger L (2021). Executive summary: Antithrombotic therapy for VTE disease: second update of the chest guideline and expert panel report. Chest.

[7] Tu T, Toma C, Tapson VF (2019). A prospective, single-arm, multicenter trial of catheter-directed mechanical thrombectomy for intermediate-risk acute pulmonary embolism: the flare study. JACC Cardiovasc Interv.

[8] Pelage JP, El Hajjam M, Lagrange C, Chinet T, Vieillard-Baron A, Chagnon S, Lacombe P (2005). Pulmonary artery interventions: an overview. Radiographics.

[9] Lafita V, Borge MA, Demos TC (2007). Pulmonary artery pseudoaneurysm: etiology, presentation, diagnosis, and treatment. Semin Intervent Radiol.

[10] Murakami T, Otomo Y, Ito T, Sato K, Ohba T (2024). Infectious pulmonary artery pseudoaneurysm secondary to a lung abscess treated with pulmonary artery coil embolization: a case report. Cureus.

[11] Barrot V, Pellerin O, Reverdito G, Sapoval M, Boeken T (2022). Ruptured pulmonary artery pseudoaneurysm treated with stent graft: case report and literature review. CVIR Endovasc.

[12] Park A, Cwikiel W (2007). Endovascular treatment of a pulmonary artery pseudoaneurysm with a stent graft: report of two cases. Acta Radiol.

[13] Munshi NH, Spinosa D, Ryan L, Butros P (2022). Endovascular repair of an iatrogenic pulmonary artery pseudoaneurysm. JACC Case Rep.

[14] Chen Y, Gilman MD, Humphrey KL (2017). Pulmonary artery pseudoaneurysms: clinical features and CT findings. AJR Am J Roentgenol.

[15] Fish A, Sailer A, Pollak J, Schlachter T (2023). Pulmonary artery pseudoaneurysms: a single-center experience of endovascular occlusion. CVIR Endovasc.

[16] Fontana F, Piacentino F, Curti M (2023). Pulmonary artery pseudoaneurysms embolization: bicentric experience and review of the literature. J Clin Med.

[17] Li FQ, Su DJ, Zhang WJ (2023). Endovascular treatment for massive haemoptysis due to pulmonary pseudoaneurysm: report of 23 cases. J Cardiothorac Surg.

